# Utilising the nasal aperture for template stabilisation for guided surgery in the atrophic maxilla

**DOI:** 10.1186/s40729-020-00221-x

**Published:** 2020-06-26

**Authors:** Pieter Onclin, Joep Kraeima, Bram B. J. Merema, Henny J. A. Meijer, Arjan Vissink, Gerry M. Raghoebar

**Affiliations:** 1 Department of Oral and Maxillofacial Surgery, University of Groningen, University Medical Centre Groningen, PO Box 30.001, NL-9700 RB Groningen, The Netherlands; 2 Department of Implant Dentistry, University of Groningen, University Medical Centre Groningen, PO Box 30.001, NL-9700 RB Groningen, The Netherlands

**Keywords:** Edentulous, Atrophic, Maxilla, Template, Implant, Accuracy, 3D VSP

## Abstract

**Background:**

Templates aim to facilitate implant placement in the prosthetically preferred position. Mucosa-supported and bone-supported templates are commonly used in the edentulous maxilla. In the atrophic maxilla (Cawood V and VI), however, these templates can be easily displaced due to a lack of supportive tissues, even in cases where anterior sites offer sufficient bone for implant placement. To assist in positioning and stabilisation, we designed a template that utilises the nasal aperture as a fulcrum to create a forced and exclusive fit. The aim of this study was to assess the clinical usability of the developed template and the corresponding implant placement accuracy in patients with edentulous atrophic maxillae. Deviations between planned and placed implant positions were measured by aligning pre- and post-operative cone beam computed tomography scans.

**Results:**

Twenty-four implants were placed in 11 patients. One template did not fit properly due to a slight undercut. All implants could be placed with good primary stability. The implants had high accuracy at the implant shoulder (global deviation 1.1 ± 0.5 mm, lateral deviation 0.8 ± 0.5 mm) and a mean angular deviation of 7.2 ± 3.4°.

**Conclusions:**

The developed surgical template offers stabilised and secure template placement in the edentulous atrophic maxilla, resulting in satisfying implant placement accuracy when using a semi-guided approach.

**Trial registration:**

Netherlands Trial Register, NL6561, registered 26 September 2017.

## Background

Surgical templates aim to aid in placing implants in the prosthetically preferred position. They can be used in a flapless approach, supported by teeth or mucosa, or in an open flap approach supported by the bone. There are several forms of (non)guidance. Non-guided means the implants are placed free-handed without the use of a template. Semi-guided templates are used to guide cavity preparation and are removed at implant insertion. Fully guided templates are used for both cavity preparation and implant insertion. When considering the use of templates for implant treatment, especially in the atrophic edentulous maxilla, clinical aspects, such as the available bone volume, are important. Cawood and Howell’s classification [1] of edentulous resorption patterns may assist in choosing the type of template.

When treating the edentulous maxilla, mucosa-supported templates (MSTs) can enable minimal invasive implant placement with mean accuracies at the implant shoulder varying from 0.8 to 1.7 mm and with mean angular deviations of 1.9 to 8.4° [2–7]. MSTs are often designed using the double scan technique, which, among other things, utilises the patient’s denture as a template bas e[8]. Since implant placement inaccuracies are mainly caused by MST positioning error, stability is crucial [2] and a safety margin around the implant of 3 mm is advised [6]. Therefore, MSTs may only be suitable for Cawood class II (post-extraction) and III (rounded ridge) cases.

An open flap approach is more appropriate for Cawood class IV (knife edge ridge) [9, 10], using bone-supported templates (BSTs). BST accuracies are similar to MST ones (with an implant shoulder deviation of 0.7–1.6 mm and a mean angular deviation of 2.4 to 4.6°) [11–13]. Using an open flap approach means any bone dehiscence can be directly noticed and resolved during surgery. The alveolar process offers a bony support which allows for less template displacement compared to a mucosa-supported template. However, the template may easily be displaced in the atrophic maxilla (Cawood class V or VI) [2, 3, 14], even though these cases may offer sufficient bone volume in the anterior maxilla. Additionally, the aforementioned high accuracies are not achievable in lower bone quality conditions [13].

To overcome template positioning problems in the atrophic maxilla, a semi-guided template was developed that utilises the nasal aperture as a fulcrum, creating a more forced and exclusive template fit, allowing for more confident and safe implant placement. To the best of our knowledge, this is the first study to utilise the nasal aperture to support a surgical template. The aim of this study was to assess the clinical usability of the developed template and corresponding implant placement accuracy in patients with edentulous atrophic maxillae.

## Methods

### Patients

All eligible patients referred between November 2017 and November 2018 to the Department of Oral and Maxillofacial Surgery of the University Medical Centre Groningen, with an extremely resorbed maxilla (Cawood V/VI) and suffering from retention and stability problems of their upper denture, were invited to participate in this prospective case series. The patients had to be at least 18 years of age, non-smoking and fully edentulous for at least 1 year; had sufficient bone volume for placement of two to four implants in the anterior maxilla; had no medical impediments for surgery; and had not had previous implant surgery in the maxilla.

### Intake procedure

After receiving information about the treatment and the study, written informed consent was obtained from each patient. The study was approved by the Medical Ethical Committee of the University Medical Centre Groningen (number 201700666). The study was registered in the Netherlands Trial Register (number NL6561). To enable 3D virtual surgical planning (VSP), patients were scanned using the double scan procedure as described by Verstreken [3]. Two-millimetre glass spheres were glued to the denture using sticky wax (Kemdent, Purton, UK). A CBCT scan (Planmeca Promax 3D Max, Planmeca, Helsinki, Finland) of both the patient wearing the denture and the denture itself was made. The following settings were used: 120 kV, 5 mA, 8 s exposure and 200 μm voxel size. The volume of interest was set from the lower part of the zygomatic arch to the maxilla.

### 3D VSP and template design

**Fig. 1:**
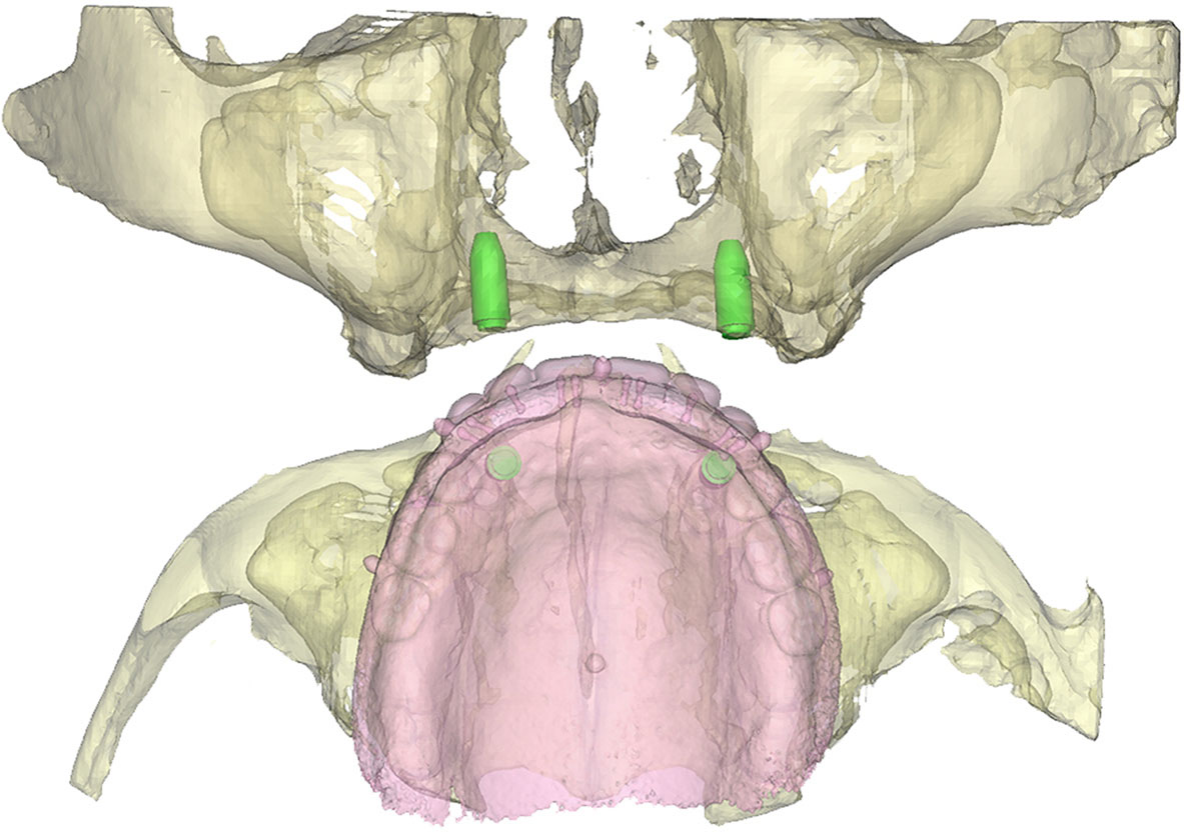
Implant planning. The images of the patient and the prosthesis are segmented, aided by the double scan method, and the implants are virtually planned in a prosthetically driven way

**Fig. 2:**
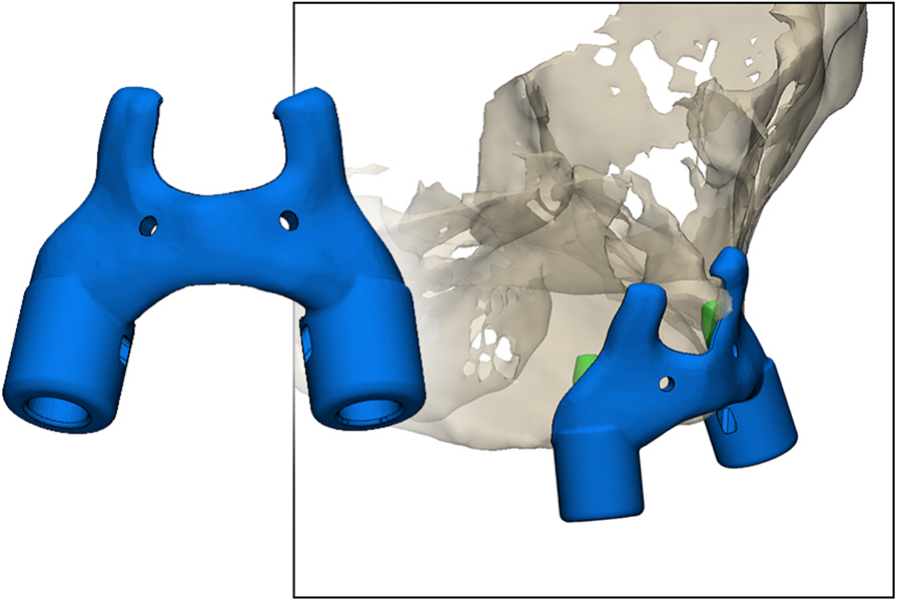
Template design. Notice the brackets in the anterior nasal aperture forcing the template into position ensuring an exclusive fit. Also note the two holes that were added to be able to get a good retention of the template. In none of our patients, however, we had to insert screws to allow for the required stability of the template as the template was stable without the use of screws

Following imaging, a 3D model of the patient including the bone and prosthesis was obtained in Proplan CMF 3.0 (Materialise, Leuven, Belgium). Two to four virtual analogues with a diameter corresponding to the implants were planned underneath the virtual denture. The surgeon (GMR) and prosthodontist (HJAM) were consulted to confirm the position of the implants (Fig. 1). Subsequently, using 3-Matic Medical 11.0 (Materialise, Leuven, Belgium), a bone-supported template was designed and partially extended to the nasal aperture (Fig. 2). Every implant location had a width compatible with the implant manufacturer’s drill sleeves, which were in close contact with the bone. The designed template was exported as a ‘Surface Tessellation Language’ (STL)-file for manufacturing using medical-grade polyamide powder for selective laser sintering (Oceanz BV, Ede, The Netherlands).

### Surgical procedure

**Fig. 3:**
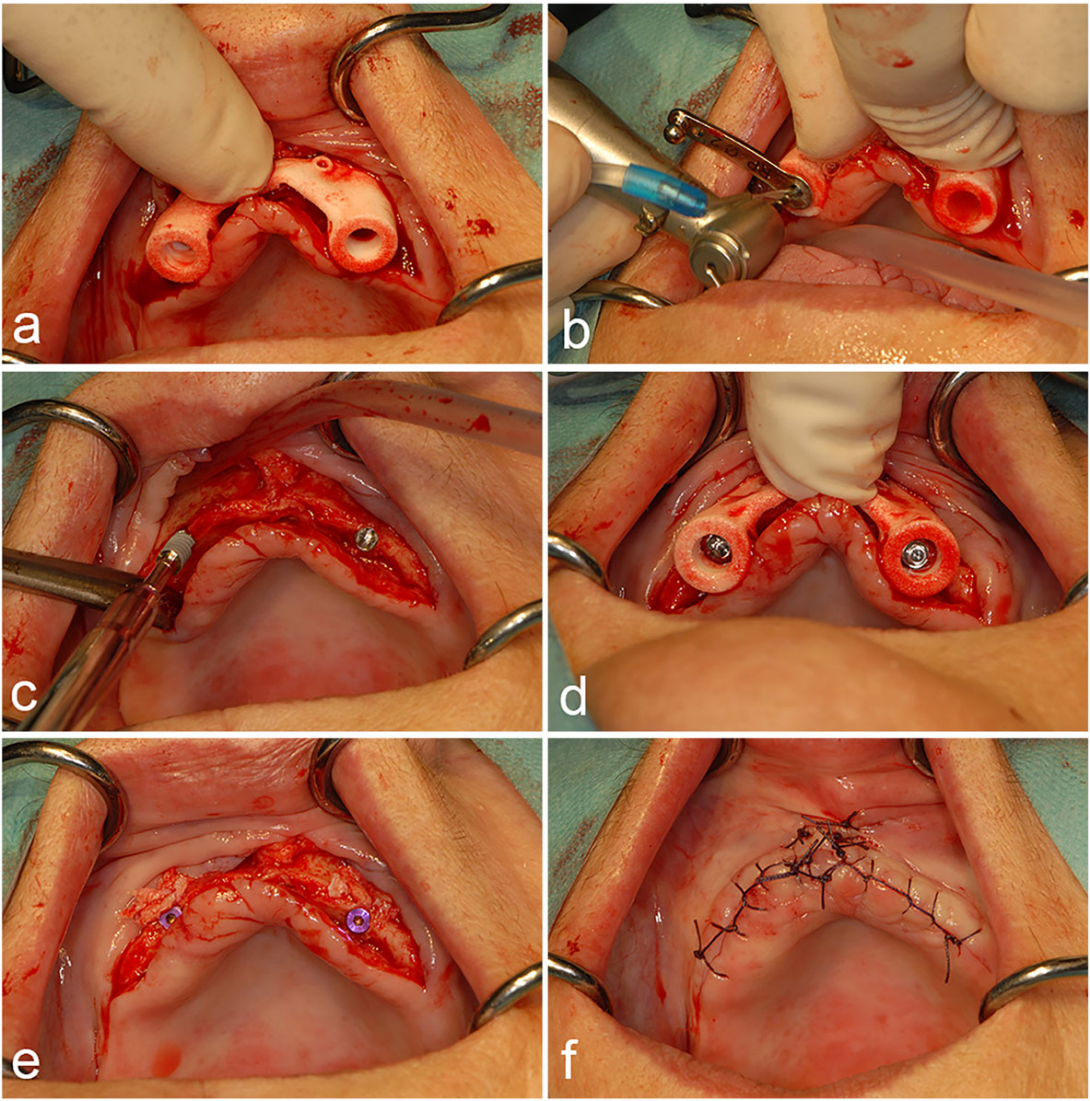
Surgical procedure. a The template is positioned by placing it on the alveolar ridge. Then, the two brackets are rotated onto the nasal notch. **b** The implant beddings are prepared using the manufacturer’s sleeves. **c** After preparation, the implants are manually placed at 45 N cm. Notice the thin atrophic alveolar crest. **d** The implants are positioned according to the template. **e** The cover screws are placed. **f** The wound is sutured with a non-resorbable material

All the patients were treated by one surgeon (GMR). The surgical procedure is shown in Fig. 3. The templates were autoclaved before use. Four patients were treated under general anaesthesia, and the other seven patients were treated with local anaesthetics (Ultracain® D-S forte, Sanofi Aventis, Gouda, Netherlands). First, the crest of the alveolar process was incised and the buccal aspect of the mucosa was reflected up to the nasal anterior aperture after raising a full thickness flap. The two template brackets were then positioned on the nasal aperture, followed by placing the template in full contact with the underlying bone. Next, consecutive diameter drill sleeves were used to guide the implant drills during osteotomy, following the manufacturer’s instructions. Then, the template was removed and the implants (Nobel Active NP 3.5 mm, Nobel Biocare®, Zurich, Switzerland) were placed with a minimum torque of 45 N cm. If present, small bone dehiscences were covered with an intra-orally harvested bone and a resorbable membrane (Bio-Gide®, Geistlich Pharma North America Inc., Princeton, USA). Lastly, after the insertion of cover screws, the flap was repositioned and sutured.

Following surgery, the template fit was recorded and the patients were instructed not to wear their denture for 2 weeks. A CBCT scan was made to check implant positioning, using similar scan settings. The sutures were removed, and the patient’s denture was adjusted and relined with a soft reline (Soft-Liner, GC, Leuven, Belgium). The patients were examined during the regular check-ups at 6 and 12 weeks. After osseointegration, an implant overdenture was made by one prosthodontist (HJAM).

### Analysis

**Fig. 4:**
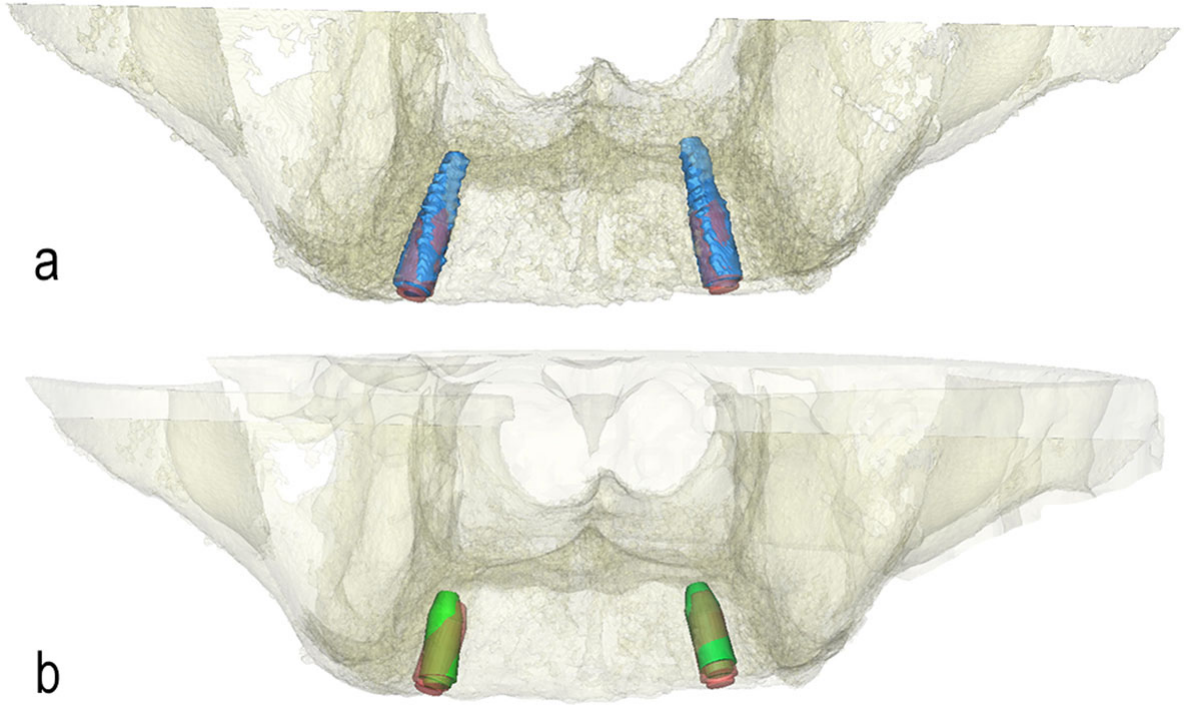
Aligning scans. **a** Virtual analogues (red) of the implants are aligned exactly with the segmented implants (blue). **b** The virtually planned and post-op datasets are aligned using the surface-based matching functions

**Fig. 5:**
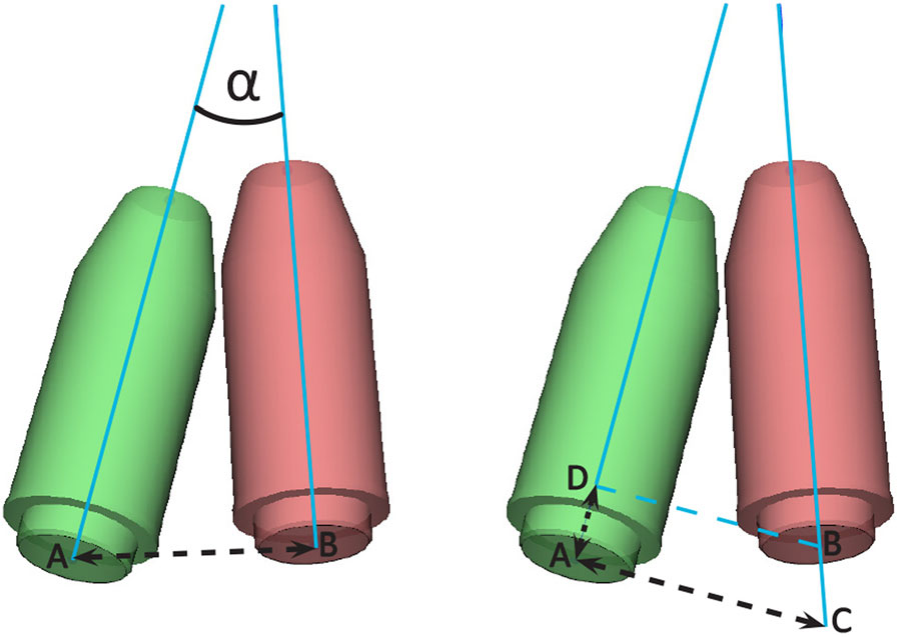
Deviation measurement. Global (point A–B), lateral (point A–C), depth (point A–D) and angular (*α*) deviations

Following segmentation of the post-op CBCT, virtual analogues of the implants were aligned exactly on the post-op CBCT data by the first observer (PO), in order to obtain identical objects representing both the planned and post-operative positions of the implants (Fig. 4a). Then, both the virtually planned and the post-op datasets were aligned using the surface-based matching functions of the Proplan software (Fig. 4b). The following deviations were measured (Fig. 5): global deviation, which is the 3D distance between the coronal centres of the planned and placed implants (point A to B); angular deviation, which is the angle between the longitudinal axes of the planned and placed implants; lateral deviation, which is the distance between the intersection of the plane perpendicular to the planned implant shoulder with the longitudinal axis of the placed implant (point C) and the coronal centre of the planned implant (point A); and depth deviation, which is the distance between the coronal centre of the planned implant (point A) and the centre point of a plane parallel to the plane perpendicular to the planned implant shoulder (point D), which intersects through the coronal centre of the placed implant (point B). The deviations between the planned and placed implants were measured in 3Matic Medical 11.0. The results were presented as descriptive statistics (Table 1).

**Table 1:** Template fit and deviations at the entry point of placed implants

Patient no.	Template fit	Implant location	Implant length (mm)	Angular deviation (°)	Global deviation (mm)	Lateral deviation (mm)	Depth deviation (mm)
1	Good	13	15	5.0	1.0	0.8	0.6
		23	15	7.7	1.2	0.6	1.0
2	Good	13	13	5.2	1.0	0.9	0.4
		23	13	4.5	0.9	0.5	0.7
3	Good	12	13	4.2	0.4	0.3	0.3
		15	13	8.9	0.4	0.2	0.4
		22	13	0.6	1.1	0.4	1.0
		25	13	4.1	0.9	0.4	0.9
4	Good	13	13	8.9	2.6	2.6	0.2
		23	13	13.0	1.9	1.9	0.1
5	Good	13	15	7.9	0.4	0.4	0.2
		23	15	8.7	0.5	0.5	0.1
6	Bad	13	15	9.3	1.1	0.8	0.7
		23	NA	NA	NA	NA	NA
7	Good	13	13	8.7	1.3	0.8	1.0
		23	13	6.2	0.9	0.6	0.6
8	Good	13	15	10.8	1.2	1.0	0.7
		23	15	8.2	1.2	0.6	1.2
9	Good	13	13	7.6	1.5	0.9	1.2
		23	13	12.5	0.4	0.3	0.2
10	Good	13	13	13.0	1.0	1.0	0.1
		23	13	1.5	2.0	0.8	1.8
11	Good	13	13	2.1	1.0	1.0	0.1
		23	13	8.0	0.7	0.6	0.3
Mean (SD)				**7.24 (3.39)**	**1.1 (0.5)**	**0.8 (0.5)**	**0.6 (0.4)**

Good fit means clinical template stability and close fit. *NA* not applicable

To examine inter-observer variation of the analysis method, a second observer (JK) repeated the virtual alignment of the analogues on the post-op CBCT data with a randomly selected sample (*n* = 5). The intra-class correlation coefficient was analysed using a two-way mixed model (absolute agreement, 95% CI) for the selected cases.

## Results

### Clinical performance

A total of 24 implants was placed in 11 patients. The implants had a length of 13 to 15 mm. All but one template had a good fit during implant placement, which means that the templates could be placed easily at the nasal fulcrum and they fit the labial aspect of the alveolar process precisely (Table 1). One template could not be placed properly on the left side. The left clamp failed to slide over the nasal aperture after placing the template on the alveolar bone. Once the left clamp was removed, the template could be properly positioned. However, the accuracy was not calculated for this implant (patient 6, implant location 23). All the other implants were placed with good primary stability, and no post-operative complications occurred. Post-operative pain was self-limiting and managed with over-the-counter pain medication. After a 3-month osseointegration period, second-stage surgery was performed; no implants were mobile so healing abutments were placed and an implant-supported maxillary overdenture was fabricated. None of the implants was lost during the follow-up period of 6 months after implant placement.

### Inter-observer variation

The intra-class coefficient between the two observers was 0.84 for global deviation, with a mean difference of < 0.1 mm, which indicates good reproducibility.

### Accuracy

Implant accuracy results are presented in Table 1. The mean deviations at the implant shoulder were 1.1 ± 0.5 mm (global deviation), 0.8 ± 0.5 mm (lateral deviation) and 0.7 ± 0.4 mm (depth deviation). The mean angular deviation of the implants was 7.2 ± 3.4°.

## Discussion

The aim of this study was to assess the clinical usability and accuracy of nasal aperture-supported templates in patients with severe maxillary bone atrophy (Cawood V and VI). Overall, the template fits were stable and secure, with satisfying implant placement accuracy.

Recent BST studies show a mean global deviation at the implant shoulder of 0.7 ± 0.4 mm to 1.6 ± 0.9 mm and a mean angular deviation of 2.4 ± 1.0° to 4.6 ± 2.6° [11–13]. These results are comparable to the current study, despite the fact that semi-guided templates were used here on, and despite the fact that only Cawood class V or VI patients were treated, while the other studies did not mention the available bone volume.

Our angular deviation was slightly higher than in other studies. Vercruyssen et al. [13] showed that a lack of guided placement did not affect angular deviation when comparing templates with semi- and full guidance. However, their results were not corrected for different types of bone density. Ozan et al. [12] compared angular deviation between groups with high and low bone density, measured using Hounsfield units. They detected significantly higher angular deviations in the low bone density group when a semi-guided template was used. This may partly explain the slightly higher angular deviations found in our study, compared to others. However, we used an implant type that allows for a cutting action at the apex during placement. This enables the surgeon to make slight changes in the angular orientation, while maintaining the right position at the implant shoulder, so bone dehiscence can be prevented. Moreover, the clinical relevance of these angular deviation values is debatable. In edentulous cases that are rehabilitated with an implant overdenture, like in this study, the prosthodontist is able to overcome such minimal deviations.

One template could not be placed properly on the left side. An unplanned undercut of the clamp during the design process may have caused this. Additionally, model segmentation is crucial for proper template fit. During segmentation, the CBCT image is converted to a 3D model by manually choosing the grey values corresponding to the bone. Incidental underestimation of the representative bone is common as demonstrated by Vercruyssen et al. [13]. This could also cause an absence of small irregularities which may interfere with proper placement during surgery. In order to reduce the influence of segmentation error, this step and the 3D virtual planning were only performed by experienced users only and discussed before initiating production. A slight offset of the template was used to compensate for any irregularities.

A known limitation in accuracy studies using CBCT is the technical steps that are needed to compare planned and placed implants. The post-operative CBCT scan needs to be segmented, which induces similar inaccuracies to those in the planning stage. Moreover, even though the post-operative scan is aligned with the pre-operative scan using a surface-based algorithm, both scans differ slightly from each other. Lastly, the titanium implants cause a scattered image, which makes the alignment of the virtual analogues to the placed implants difficult. Since this last step is susceptible to inter-observer variation, it was validated in our study by repeating the alignment and measurement by a second observer (JK). While most studies did not validate this step, Vieira et al. [5] tested intra-observer reproducibility through Cohen’s kappa analysis. This resulted in a kappa value of 0.72, which corresponds to a substantial agreement, comparable with our findings.

Although other studies tried to define a mesio-distal and bucco-lingual plane of deviation, because they seem more clinically relevant than lateral deviation, we consciously did not apply these techniques. Defining the planes requires human interpretation, which again affects the validity of the data. The same applies for the defined depth deviation. However, since our study did not control the depth with guided placement, the depth axis was extracted from the horizontal implant deviation to give a more valid outcome and interpretation.

Even though technology is improving rapidly, it seems unrealistic to think that accuracy could improve further. Improving the application of guided surgery, as in the current study, may be more relevant. Cost-effectiveness is also underexposed in current literature. Younes et al. [15] state that templates can be justified in partially edentulous cases because they can prevent prosthetic cementation. The same may apply for MSTs because they improve the patient’s experience through a time-saving treatment and lower post-operative complaints than in a flapped approach [16]. On the other hand, the use of BST may prevent the need for extensive and expensive augmentation surgery because implants can even be planned and placed with satisfying accuracy in the atrophic maxilla, like in this study which may result in lower post-operative complaints and lower costs.

## Conclusion

It can be concluded that the developed surgical template offers stable and secure template placement in the edentulous atrophic maxilla and satisfying implant placement accuracy when using a semi-guided approach.

## Data Availability

The datasets used and/or analysed during the current study are available from the corresponding author on reasonable request.

## Abbreviations

BST: Bone-supported templates
CBCT: Cone-beam computer tomography
MST: Mucosa-supported templates
STL: Surface Tessellation Language
VSP: Virtual Surgical Planning
